# Participation of the oviductal s100 calcium binding protein G in the genomic effect of estradiol that accelerates oviductal embryo transport in mated rats

**DOI:** 10.1186/1477-7827-9-69

**Published:** 2011-05-23

**Authors:** Mariana Ríos, Alexis Parada-Bustamante, Luis A Velásquez, Horacio B Croxatto, Pedro A Orihuela

**Affiliations:** 1 Unidad de Reproducción y Desarrollo, Facultad de Ciencias Biológicas, Pontificia Universidad Católica de Chile, Chile; 2 Laboratorio de Inmunología de la Reproducción, Facultad de Química y Biología, Universidad de Santiago de Chile, Chile; 3 Centro para el Desarrollo en Nanociencia y Nanotecnología-CEDENNA, Santiago, Chile; 4 Millennium Institute for Fundamental and Applied Biology, Santiago, Chile

## Abstract

**Background:**

Mating changes the mechanism by which E2 regulates oviductal egg transport, from a non-genomic to a genomic mode. Previously, we found that E2 increased the expression of several genes in the oviduct of mated rats, but not in unmated rats. Among the transcripts that increased its level by E2 only in mated rats was the one coding for an s100 calcium binding protein G (s100 g) whose functional role in the oviduct is unknown.

**Methods:**

Herein, we investigated the participation of s100 g on the E2 genomic effect that accelerates oviductal transport in mated rats. Thus, we determined the effect of E_2 _on the mRNA and protein level of s100 g in the oviduct of mated and unmated rats. Then, we explored the effect of E_2 _on egg transport in unmated and mated rats under conditions in which s100 g protein was knockdown in the oviduct by a morpholino oligonucleotide against s100 g (s100 g-MO). In addition, the localization of s100 g in the oviduct of mated and unmated rats following treatment with E_2 _was also examined.

**Results:**

Expression of *s100 g *mRNA progressively increased at 3-24 h after E2 treatment in the oviduct of mated rats while in unmated rats *s100 g *increased only at 12 and 24 hours. Oviductal s100 g protein increased 6 h following E_2 _and continued elevated at 12 and 24 h in mated rats, whereas in unmated rats s100 g protein increased at the same time points as its transcript. Administration of a morpholino oligonucleotide against *s100 g *transcript blocked the effect of E_2 _on egg transport in mated, but not in unmated rats. Finally, immunoreactivity of s100 g was observed only in epithelial cells of the oviducts of mated and unmated rats and it was unchanged after E2 treatment.

**Conclusions:**

Mating affects the kinetic of E2-induced expression of s100 g although it not changed the cellular localization of s100 g in the oviduct after E_2 _. On the other hand, s100 g is a functional component of E2 genomic effect that accelerates egg transport. These findings show a physiological involvement of s100 g in the rat oviduct.

## Background

In mammals, the transport of the gametes, fertilization or preimplantation development depends on the oviduct functions [1]. In this context, the participation of ovarian hormones estradiol (E_2_) or progesterone (P) is crucial for the successful of these events. Oviduct malfunction may result in tubal ectopic pregnancy that still remains a potentially life-threatening condition in women [2]. Therefore, elucidation of the cellular and molecular mechanisms by which E_2 _and P regulate oviductal egg transport are essentials to fully understand the physiology of the tubal function.

In the rat, the duration of oviductal egg transport is dependent on ovarian hormones and mating-associated signals [3-5]. A single injection of E_2 _on day 1 of the cycle (unmated) or pregnancy (mated) shortens oviductal transport of eggs from the normal 72-96 h to less than 24 h [4,6]. However, E_2 _accelerates egg transport to the uterus through intraoviductal genomic pathways in mated rats and through nongenomic pathways in unmated rats [3,7]. Interestingly, both pathways require activation of estrogen receptors (ER) [8,9]. The intraoviductal nongenomic-signalling pathway of E_2 _has been well established and involves conversion of E_2 _to 2-methoxyestradiol (2ME) and sequential activation of cAMP-PKA and PLC-IP3 signalling pathways [5,8,10]. In contrast, little is known about the components of the E_2 _genomic signaling that accelerates embryo transport in the rat. Early works have shown that antagonists of Endothelin receptor type A or B inhibited the effect of E_2 _on embryo transport [11] while Connexin 43 uncouplers blocked the increase in the instant velocity of microsphere movement induced by E_2 _in mated rats [12]. Thus, the E_2 _genomic pathway involves participation of endothelin receptors (ETR) and functional integrity of gap junctions in the oviduct.

Previously, we have found by microarray analysis that E_2 _increased the expression of several genes in the oviduct of mated rats, but not in unmated rats [13]. These genes were s100 calcium binding protein G (*s100 g*), adenosine monophosphate deaminase 3 (*Ampd3*), cysteine rich protein 61(*Cyr61*) and tumour necrosis factor induced protein (*Tnfip6*) that are involved in remodelation of extracellular matrix, regulation of energetic metabolism or calcium transport in normal tissues or tumour cells [14-16]. In order to identify whether these genes are functional components of the E_2 _genomic pathway that accelerates embryo transport in mated rats, we first compared the mRNA level of these four transcripts between oviducts of unmated and mated rats treated with E_2_. We reasoned that genes whose level of expression increases in response to E_2 _in mated, but not in unmated rats are good candidates to further exploration. The results oriented us towards a possible involvement of *s100 g *transcript previously described as Calbindin-D9k. s100 g belongs to a family of intracellular proteins having high affinity for calcium and is expressed in a variety of mammalian tissues, i.e., intestine, uterus, kidney and bone [17-19] including the female reproductive tract [20,21]. Furthermore, estrogens up-regulates the expression of the mRNA [18] and protein [18] of s100 g in the uterus. Moreover, s100 g may control motile activity of smooth muscle through regulation of intracellular calcium level in the rat uterus [18]. Therefore, we investigated the participation of s100 g on the E_2 _genomic effect that accelerates oviductal embryo transport. For this purpose, we determined the time-course of the effect of E_2 _on the mRNA and protein level of s100 g in the oviduct of mated and unmated rats. Then, we explored the effect of E_2 _on egg transport in unmated and mated rats under conditions in which s100 g protein was knockdown in the oviduct by a morpholino oligonucleotide against *s100 g *(s100 g-MO). Finally, the localization of s100 g in the oviduct of mated and unmated rats following treatment with E_2 _was also examined.

## Methods

### Animals

Locally bred Sprague-Dawley rats were used. Animals were kept under controlled temperature (21-24°C) and lights were on from 07:00 to 21:00 h. Water and pelleted rat chow was supplied *ad libitum*. Daily vaginal smears were used to verify cycle regularity [22]. Females weighing 200-220 g were selected from among those having 4-day estrous cycles. Females in proestrus were either kept isolated or caged with fertile males. The following day (estrus) was designated as Day 1 of the cycle (C1) in the first instance and Day 1 of pregnancy (P1) in the second, provided spermatozoa were found in the vaginal smear of the later. The protocols on animal manipulation have been approved by the Ethical Committees of our National Fund of Science (CONICYT-FONDECYT 1080523).

### Treatments

#### Systemic administration of E_2_

Rats on C1 or P1 were injected s.c. with 10 mu μg of E_2 _as a single dose dissolved in 0.1 mL of propylene glycol. Control rats received propylene glycol as the vehicle.

#### Local administration of morpholinos (MOs)

The following MOs were used: s100 g-MO, 5'CGC TCA TTT TTC TGT GCT GCT TGC T 3'; an irrelevant MO (standard control MO) 5'CCT CTT ACC TCA GTT ACA ATT TAT A 3'; and a Fluoresceinated -labeled standard control MO (FITC-MO). All MO were purchased from Gene Tools, LLC, Philomarth, OR. The MOs were prepared at a stock concentration of 10 mM in sterile water. For intraoviductal administration (i.o), 0.2 mu μl of Endo-Porter (non-toxic delivery mechanism from Gene Tools) was mixed with 2.8 mu μl of MO stock (28 nmoles). The MO/Endo-Porter mixture was vortexed and incubated for 20 min at room temperature, immediately prior to injection into each oviduct.

### Animal surgery

Intraoviductal administration of MOs was done in the morning of C1 or P1 using a surgical microscope (OPMI 6-SDFC; Zeiss, Oberkochen, Germany) as previously described [3,7]. Since ovulation was completed at this time point, this treatment did not affect the number of oocytes that ovulated.

### Assessment of egg transport

Animals were euthanized 24 hours after different treatments, and their oviducts were flushed individually with saline. Each flushing was examined under low-power magnification (25×). The number of eggs in both oviducts was recorded as a single datum. Attempts to recover eggs from the uterus and vagina with or without placing ligatures in the uterine horns have shown that the reduction in the number of oviductal oocytes following treatment with E_2 _corresponds to premature transport to the uterus [6]. Thus, we refer to it as E_2_-induced acceleration of oviductal transport.

### Analysis of MO Uptake

Animals were euthanized 5 hours after i.o. administration of FITC-MO or standard control MO and their oviducts were excised and frozen in Tissue Freezing Medium (Electron Microscopy Sciences, Washington, PA). Five-micrometer thick frozen sections were fixed in ethanol 70% and mounted with Fluoromount G (Electronic Microscopy Science, Washington, PA), and then analyzed under an Optiphot Epifluoresence Microscope (Olympus, Middlebush, NJ).

### Real-Time Polymerase Chain Reaction (qPCR)

Animals were euthanized and their oviducts were collected and flushed with saline. Total RNA was isolated using Trizol Reagent (Invitrogen Co., California, CA) and 1 mu μg of total RNA of each sample (2 oviducts from 1 rat) was treated with Dnase I Amplification grade (Invitrogen). The single-strand cDNA was synthesized by reverse transcription using the Superscript III Reverse Transcriptase First Strand System for RT-PCR (Invitrogen), according to the manufacturer's protocol. The Light Cycler instrument (Roche Diagnostics, GmbH Mannheim, Germany) was used to quantify the relative transcript level of *s100 g, Ampd3, Cyr61 *and *Tnfip6 *while *Gapdh *was chosen as the housekeeping gene for load control because we have previously demonstrated that E_2 _or pregnancy did not affect its expression [9,12]. The SYBR^® ^Green I double-strand DNA binding dye (Roche Diagnostics) was the reagent of choice for these assays. The primers used in qPCR are listed in Table 1. All real time PCR assays were performed in duplicate. The thermal cycling conditions included an initial activation step at 95°C for 25 min, followed by 40 cycles of denaturizing and annealing-amplification (95°C for 15 sec, 60°C for 15 sec and 72°C for 30 sec) and finally one cycle of melting (95° to 60°C). To verify specificity of the product, amplified products were subject to melting curve analysis as well as electrophoresis, and product sequencing was performed to confirm identity using an ABI Prism 310 sequencer. The expression of *s100 g, Ampd3, Cyr61, or Tnfip6 *were determined using the equation: Y = 2^-ΔCp ^where Y is the relative expression, Cp (crossing point) is the cycle in the amplification reaction in which fluorescence begins to be exponential above the background base line, -∆Cp is the result of subtracting Cp value of *s100 g, AmpD3, Cyr61, or Tnfip6 *from Cp value of *Gapdh *for each sample. In order to simplify the presentation of the data the relative expression values were multiplied by 10^3^[23].

**Table 1:** Primer sequences of the transcripts for *s100 g, cyr61, tnfip6, ampd3 *and gapdh

Gene	Sense	Antisense	Length of band
*s100 g*	GGCAGCACTCACTGACAGC	CAGTAGGTGGTGTCGGAGC	307 bp
*cyr61*	ATCTACCAGAACGGGGAGAG	TATTAACTCCACCTCCGAGG	253 bp
*tnfip6*	TGACAGTTATGACGATGTCC	GCCTTGATTGGATTTAGGTGC	167 bp
*ampd3*	CACCCTATGACATGCCTGAG	CAAAGAGAACACCTCCCTGC	320 bp
*gapdh*	ACCACAGTCCATGCCATCAC	TCCACCACCCTGTTGCTGTA	498 bp

### Western blot

Animals were euthanized and their oviducts were collected and flushed with saline. Total proteins were isolated, resolved by electrophoresis and electroblotted onto nitrocellulose membranes as previously described [8]. Nitrocellulose blots were blocked by incubation overnight at 4°C in TTBS (100 mM Tris/HCl pH 7.5, 150 mM NaCl and 0.05% v/v Tween 20) containing 5% nonfat dry milk. Afterward, blots were incubated with anti-rat antibodies for 1 h with a rabbit anti-rat s100 g (Swant, Bellinzona, Switzerland) or with a mouse anti-rat β-actin (clone JLA20, Calbiochem, La Jolla, CA) as load control because expression level does not change in the rat oviduct after E_2 _treatment [12] in 1:2500 or 1:5000 dilutions, respectively. Blots were rinsed 5 times for 5 min each in TBS (100 mM Tris/HCl pH 7.5, and 150 mM NaCl) and were incubated for 2 h in TTBS containing 1:5000 dilution of goat anti-rabbit or anti-mouse IgG horseradish peroxidase conjugate (Chemicon International, Temecula, CA). The horseradish peroxidase activity was detected by enhanced chemiluminescence using Western Lighting Chemiluminescence Reagent Plus (Perkin Elmer Life Sciences, Boston, MA). Negative controls consisting of oviductal samples without anti-s100 g or anti-β-actin were included. Protein extracts from immature rat uterus (18 days old) treated with E_2 _for 3 days were used as positive controls [24].

### Immunohistochemistry

Animals were euthanized and their oviducts were fixed in Bouin's solution overnight. Paraffin embedded samples cut in 6 mu μm serial sections were deparaffinized, rehydrated, and boiled in 10 mM citrate buffer (pH 6.0), for antigen retrieval. Sections were then rinsed in phosphate-buffered saline (PBS) and endogenous peroxidase was blocked by incubation with 3% v/v H_2_O_2 _for 20 minutes. After rinsing with PBS, sections were incubated with 1% w/v Bovine Serum Albumin (BSA) in PBS at room temperature to prevent unspecific binding, and then incubated with anti-rat s100 g antibody 1:100 (Swant, Bellinzona, Switzerland) in a humidified chamber overnight. The slides were washed three times for 5 min with PBS and incubated for 1 h with blocking buffer containing goat anti-rabbit IgG conjugated to horseradish peroxidase 1:1000. The antigen-antibody complexes were visualized using 3,3'-diaminobenzidine tetrahydrochloride (DAB)/Metal concentrate (Pierce, Rockford, IL). All sections were lightly counter-stained with hematoxylin (Sigma-Aldrich), dehydrated, coverslipped, and observed under a phase contrast microscope (Optiphot-2, Nikon, Japan) and photographed with a digital camera (CoolPix 4500, Nikon, Japan). The specificity of immunoreactivity was assessed by omitting the first antibody or replacing it by preimmune serum. Negative controls showed no specific immunoreactivity.

### Statistical Analyses

The results are presented as mean ± SE. Overall analysis was carried out using the Kruskal-Wallis test, followed by the Mann-Whitney test for pairwise comparisons when overall significance was detected. The actual N value in experiments to determine the effects of drugs on oviductal egg transport is the total number of rats used in each experimental group.

## Results

### Estradiol up-regulates the expression of *s100 g *and *Ampd3 *transcripts in the oviduct of mated but not of unmated rats

Here we determined the mRNA level of *s100 g, Ampd3, Cyr61 *and *Tnfip6 *in the oviducts of mated and unmated rats by quantitative RT-PCR. Rats on C1 (N = 5) or P1 (N = 5) were treated with E_2 _or vehicle and 3 h later oviducts were excised and their total RNA were processed by Real Time PCR.

**Figure 1:**
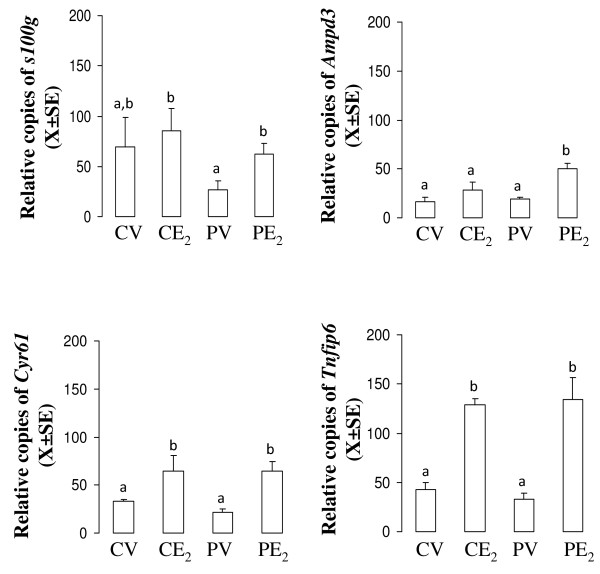
**Expression of *s100 g, Ampd3, cyr61 *or *Tnfip6 *in unmated (C) or mated (P) rat oviducts treated with E_2_**. Real-time quantitative RT-PCR analysis was done 3 hours after treatment with 10 mu μg of E_2 _or vehicle (V). N = 5 animals per group. Different letters are significantly different from each other within each graph. a ≠ b, p < 0.05.

Figure 1 shows that all four transcripts increased their mRNA level after E_2 _treatment in mated rats, but *Cyr61 *and *Tnfip6 *also increased in unmated animals. Thus, we choose *s100 g *for further exploration, leaving *Ampd3 *for future analysis.

### Time-course of the effect of E_2 _on the mRNA and protein level of s100 g in the oviducts of mated and unmated rats

This experiment was designed to establish the level of s100 g mRNA and protein at different times following E_2 _treatment. Rats injected with 10 mu μg of E_2 _or vehicle on C1 (N = 5) or P1 (N = 5) were sacrificed at 3, 6, 12 or 24 hours after treatment and the oviducts were collected to measure the level of *s100 g *mRNA by Real Time PCR. Five rats were used for each time point. Other rats on C1 (N = 3) or P1 (N = 3) were treated with E_2 _10 mu μg and 0, 3, 6 12 or 24 hours after treatment the oviducts were excised and processed to determine the level of s100 g protein by western blot.

**Figure 2:**
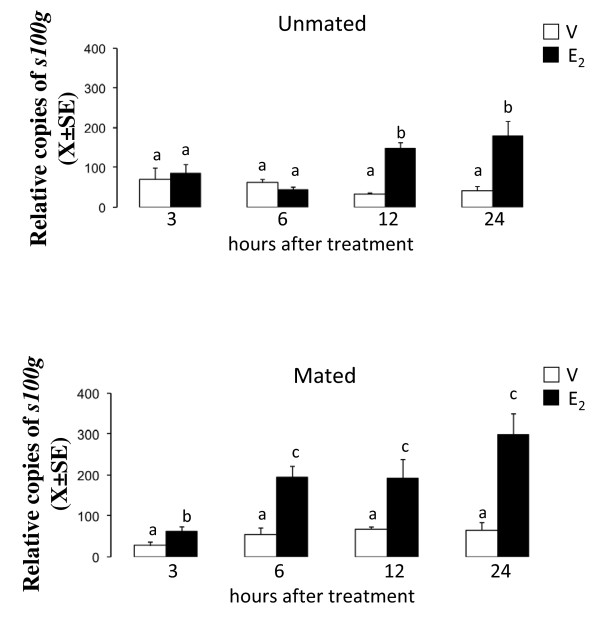
**Time course of *s100 g *mRNA level in the rat oviduct after estradiol (E_2_) treatment**. Unmated and mated rats were treated with 10 mu μg of E_2 _or vehicle and 3, 6, 12 or 24 hours later, s100 g mRNA level was determined using real-time quantitative RT-PCR. N = 5 animals per group. Different letters are significantly different from each other within each graph. a ≠ b, p < 0.05.

**Figure 3:**
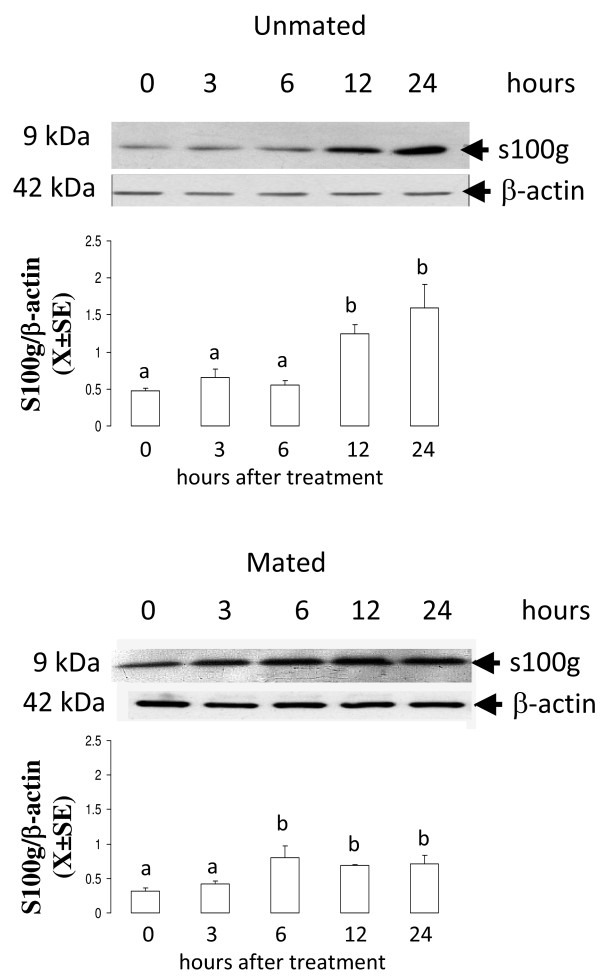
**Time course of s100 g protein level in the rat oviduct after treatment with estradiol (E_2_)**. Unmated and mated rats were treated with 10 mu μg of E_2 _or vehicle (V) and 3, 6, 12 or 24 hours later s100 g protein level was determined using immunoblotting analysis. N = 3 animals per group. Different letters are significantly different from each other within each graph. a ≠ b, p < 0.05.

*s100 g *transcript showed progressively increasing levels in response to E_2 _throughout all time points examined in mated rats while in unmated rats *s100 g *increased only at 12 and 24 hours after treatment (Figure 2). On the other hand, s100 g protein increased 6 hours after treatment with E_2 _and continued elevated at 12 and 24 hours in mated rats, whereas in unmated rats s100 g protein increased at the same time points as its transcript (Figure 3).

### Assessment of MOs uptake

In order to confirm that oviductal cells took up MOs, 3 rats on C1 were injected with 3 mu μl of FITC-MO/Endo Porter mixture into one oviduct and with Endo-Porter alone in the contralateral side. Five hours later oviducts were collected, frozen and sectioned for examination in a fluorescence microscope.

**Figure 4:**
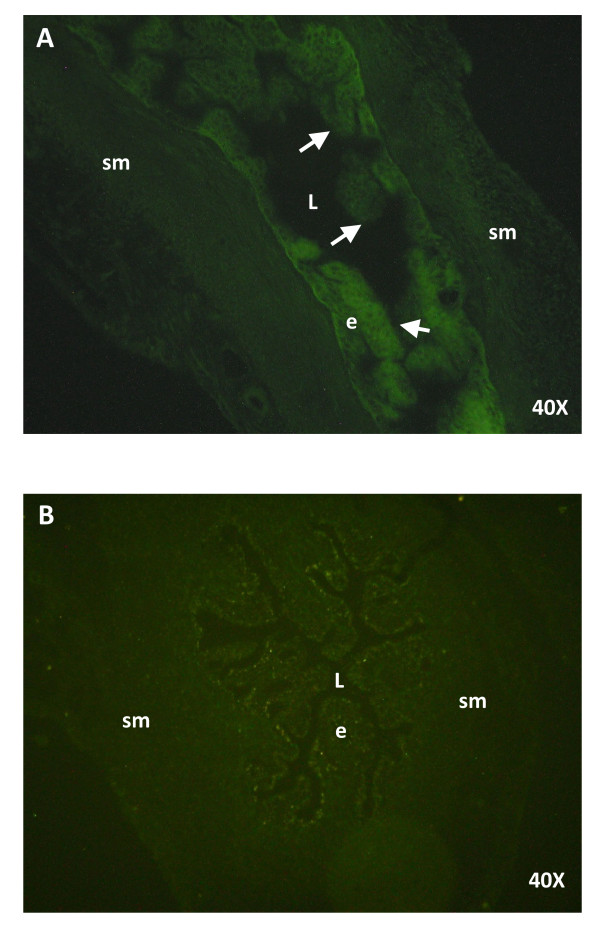
**Cellular uptake of the FITC-MO in the rat oviduct**. Photomicrographs of rat sections after 5 hours of intraoviductal injection of FITC-MO/Endo Porter mixture (A) or Endo-Porter alone (B). Note that arrows point strong green fluorescence representing cellular uptake of FITC-MO mainly in the luminal epithelium demonstrating penetration of MO in these cells *in vivo*. e = epithelium, sm = smooth muscle, L = lumen.

Strong fluorescence representing cellular uptake of FITC-MO was observed in the luminal epithelium demonstrating penetration of MO in these cells *in vivo*. No fluorescence was observed in the contra lateral oviduct, which was treated with vehicle alone (Figure 4).

### Effect of intraoviductal administration of s100 g-MO on E_2_-induced egg transport acceleration in mated and unmated rats

This experiment was designed to determine whether i.o. administration of s100 g-MO can inhibit acceleration of oviductal egg transport induced by E_2_. Rats on C1 (N = 5) or P1 (N = 7) were injected i.o. with MO/Endo-Porter mixture (s100 g-MO or standard control MO) and three hours later 10 mu μg of E_2 _was injected s.c. This dose of E_2 _accelerates oviductal transport consistently in mated and unmated rats [3,6]. A control group was included that was treated with standard control MO and the vehicle of E_2_. Twenty-four hours after i.o. administration the animals were autopsied and the number of eggs was determined as described above. To confirm that s100 g-MO effectively knockdown expression of s100 g protein in the oviduct, other rats on C1 or P1 were i.o. injected with s100 g-MO or standard control MO and 3 hours later E_2 _10 mu μg or vehicle was injected s.c to these rats. Twenty-four hours after i.o. injection oviducts were obtained to determine s100 g level by western blot.

**Figure 5:**
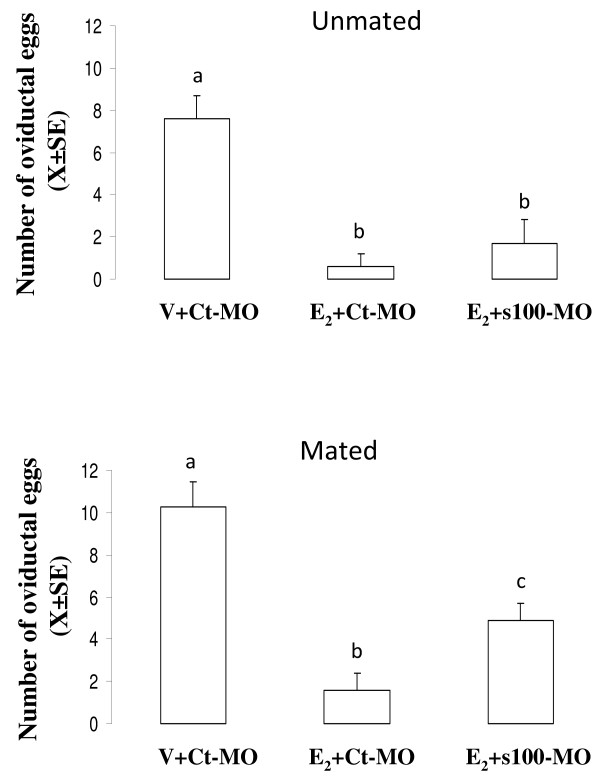
**Effect of s100 g-MO on E_2_-induced egg transport acceleration**. Number of eggs recovered from oviducts of unmated and mated rats 24 hours after intraoviductal (i.o.) administration of s100 g-MO, standard control-MO (Ct-MO) or vehicle (V) followed 3 hours later by a s.c. injection of 10 mu μg of estradiol (E_2_) or V. The number of animals per group was 5 in unmated and 7 in mated rats. Different letters are significantly different from each other within each graph. a ≠ b ≠ c, p < 0.05.

The mean number of eggs recovered from the oviducts of the control group (vehicle of E_2 _+ standard control MO) was 7.6 ± 1,1 and 10.2 ± 1.2 in unmated and mated rats, respectively. As expected, E_2 _reduced the number of oviductal eggs in unmated rats (0.6 ± 0.6) and in mated rats (1.6 ± 0.8). Local administration of s100 g-MO partially blocked the effect of E_2 _in mated, but not in unmated rats, 4.9 ± 0.8 and 1.7 ± 1.1 respectively (Figure 5). On the other hand, no changes in the rate of preimplantation embryo development were noticed in the oviducts from mated rats treated with the morpholino s100 g- MO (data not shown).

**Figure 6:**
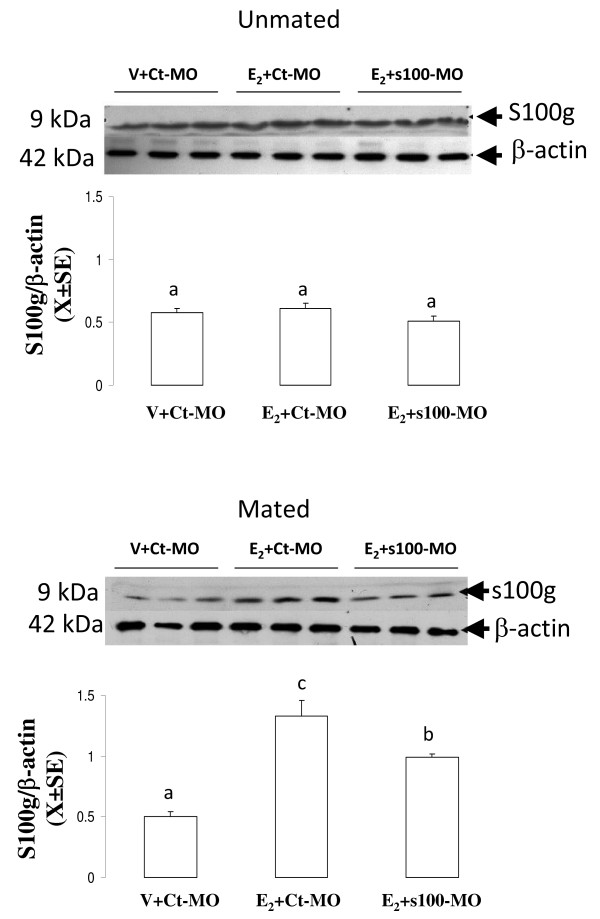
**Effect of s100 g-MO on oviductal s100g**. Level of s100 g protein in the oviduct of unmated and mated rats following intraoviductal (i.o.) administration of s100 g-MO, standard control-MO (Ct-MO) or vehicle (V) followed 3 hours later by a s.c. injection of 10 mu μg of estradiol (E_2_) or V. s100 g protein level was determined using immunoblotting analysis. N = 3 animals per group. Different letters are significantly different from each other within each graph. a ≠ b ≠ c, p < 0.05.

Intraoviductally administration of s100 g-MO partially reduced the increase of s100 g protein level induced by E_2 _in mated rats while s100 g-MO had no effect in unmated rats, as the level of s-100 g 24 hours after E_2 _was unchanged in comparison with vehicle-treated rats (Figure 6).

### Localization of s100 g protein in the oviduct of unmated and mated rats treated with E_2_

Here we determined whether mating or E_2 _treatment changes localization of s100 g protein in the oviduct. Rats injected with 10 mu μg of E_2 _on C1 (N = 3) or P1 (N = 3) were sacrificed at 12 hours after treatment and their oviducts were collected and fixed in Bouin's solution and processed by immunohistochemistry as described above.

**Figure 7:**
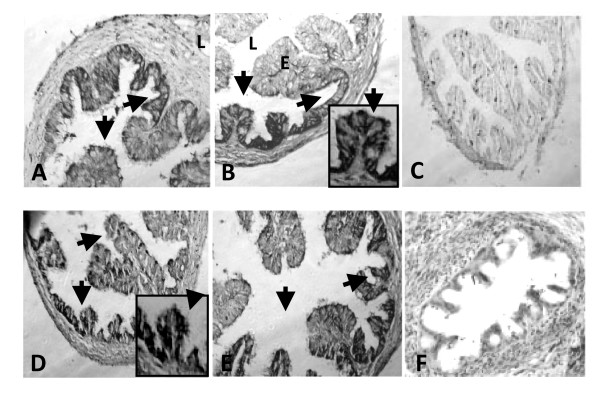
**Cellular localization of s100 g in E_2_-treated rat oviducts**. Photomicrographs of unmated (A-C) and mated (E-F) rat oviducts 12 h after injecting vehicle (A, D) or E_2_(B, E). Arrows point to immunoreactivity of s100 g present only in epithelial cells. The specificity of immunoreactivity was confirmed by omitting the first antibody or replacing by preimmune serum (C, F). Insets in B and D show higher magnification of s100 g immunoreactivity in epithelial cells.

s100 g immunoreactivity was observed only in epithelial cells of the oviducts of C1 and P1 and it was unchanged following E_2 _treatment (Figure 7).

## Discussion

Mating changes the mechanism of action by which E_2 _regulates oviductal egg transport, from a non-genomic to a genomic mode. This changes in pathways has been denominated "intracellular path shifting" (IPS) and reflects a functional plasticity in well-differentiated cells. Although, microarray analysis showed that *s100 g, Ampd3, Cyr6*1 and *Tnfip6 *transcripts increased their level only in the E_2_-treated oviducts of mated, but not in unmated rats [13] confirmation by quantitative RT-PCR demonstrated that *Cyr61 *and *Tnfip6 *increased their level in response to E_2 _in both group of rats. Therefore, we consider that *s100 g *and *Ampd3 *were only up-regulated by E_2 _in the oviducts of mated rats while *Cyr6*1 and *Tnfip6 *are up-regulated in both physiological conditions. On the other hand, these findings show for the first time evidence that E_2 _regulates expression of *Ampd3, Cyr61 *and *Tnfip6 *in the mammalian oviduct and that mating-associated signals stimulate the response of *s100 g *and *Ampd3 *to E_2 _in the rat oviduct. However, the functional relevance of *Ampd3, Cyr61 *and *Tnfip6 *in the oviductal physiology was not explored in this work.

Despite of *s100 g *and *Ampd3 *genes are potential candidates to participate in the intraoviductal E_2 _genomic effect that accelerates egg transport in mated rats we elected explore the participation of s100 g because it is known that is regulated by E_2 _in several mammalian tissues including female reproductive tract [20,25]. According with this, we found that mRNA and protein level of s100 g increased in response to E_2 _in the oviducts of mated and unmated rats. However, the effect of E_2 _was evidenced much earlier in mated rats than in unmated showing that mating-associated signals changes the kinetic of transcription or translation of s100 g. Estradiol exerts its effects after binding to ER in the nucleus, inducing a conformational change and relocation of co-regulators proteins that permits the association of the E_2_-ER complex to estrogens-responsive elements (ERE) in the DNA [26]. In the rat, the regulation of s100 g is mediated by an ERE located at the first intron of this gene [27]. The mechanism of distinct regulation of oviductal s100 g between mated and unmated rats is not clear. Probably, mating removes some co-regulator proteins that prevent the transcription of s100 g or there are differences in the stability of the s100 g mRNA or protein in the oviductal cells of mated and unmated rats, but all of this needs further exploration. On the other hand, rat oviductal level of s100 g throughout the estrous cycle or early pregnancy remains undetermined.

The administration of s100 g-MO directly into the oviductal lumen revealed that E_2_-induced expression of s100 g protein is essential for the E_2 _genomic pathway that accelerates oviductal embryo transport. The effect of s100 g-MO on accelerated egg transport was only partial in accordance with the fact that expression of the s100 g protein in the oviduct of mated rats was partially blocked by the morpholino. This may attributable to insufficiency of the dose administered-a dose chosen because higher doses could not be dissolved in the small volume that can be injected to the oviductal lumen. Furthermore, On the other hand, local injection of s100 g-MO to unmated rats neither affected the E_2_-induced egg transport acceleration nor the expression of s100 g protein in the oviduct. In contrast to previously observed (Figure 3) E_2 _did not increase the level of s100 g protein in the oviduct of unmated rats 24 h after treatment with standard control MO (Figure 6). This may be explained because in this particular group of rats, oviductal cells were more affected or damaged by the intraoviductal injection blunting the effect of E_2 _on expression of s100 g in the oviduct. Therefore, we cannot discard the participation of s100 g in the E_2 _non-genomic pathway that accelerates egg transport in unmated rats. Probably, others routes of administration of the morpholino would be required in unmated rats, but this was not done.

Although, it has been shown that in the mouse endometrial s100 g is critical for embryo implantation [25] the role of oviductal s100 g in the embryo development is unknown. Since, we did not notice any change in the development of early embryos flushed out of the oviducts from mated rats treated with E_2 _and the morpholino s100 g- MO we could speculate that morpholino s100 g did not affect preimplantation embryo development.

Previous works have shown that s100 g protein was localized exclusively in the epithelial cells of the rat oviduct [18]. Here, we report that mating did not affect the cellular localization of this molecule as well as the response to E_2 _suggesting that the role of s100 g in the E_2 _genomic pathway is not explained by a change in the localization of s100 g in the oviduct. Since the proportion between ciliated and secretory cells varies in the oviductal epithelial cells it is probable that mating-associated signals could have acted differentially on these two types of cells. Ultrastructural studies using immunoelectron microscopy needs to be done to resolve this issue.

In other tissues s100 g enhance Ca^2+ ^transport and increase in the cells capacity to store Ca^2+^[28,29]. In the uterus, s100 g may be involved in controlling myometrial activity related to regulation of the Ca^2+ ^level [20,28]. Furthermore, a role of endometrial s100 g in the embryo implantation was previously reported [25]. Since, E_2 _genomic pathway that control oviductal egg transport requires participation of calcium-dependent molecules such as ETR and Cx43 [11,12] we postulate that oviductal s100 g signalling initiated in the epithelial cells could modulate expression or activity of ETR and Cx43 to regulate contractile activity in the oviduct necessary for the embryo movement.

## Conclusions

Mating changes the kinetic of E_2_-induced expression of s100 g in the oviduct although cellular localization of s100 g was not affected by mating following E_2 _treatment. Furthermore, the E_2 _intraoviductal genomic pathway that accelerates embryo transport in mated rats requires functional participation of s100 g. These findings provide evidence of a physiological involvement of s100 g in the rat oviduct.

## Competing interests

The authors declare that they have no competing interests.

## Authors' contributions

MR participated in the design of the study, performed the sampling of the animals, carried out the Real-Time PCR, intraoviductal injections of drugs and assessment of the egg transport. APB collaborated in the design of the studies of the western blot and immunohistochemistry of s100 g. LV and HBC participated in planning experiments and contributed to drafting the manuscript. PAO participated in the design of the study, in directing and completing all experimental analysis and in writing the manuscript. All authors have read and approved the final manuscript.
